# Factors Associated with Influenza Vaccination Uptake among U.S. Adults: Focus on Nativity and Race/Ethnicity

**DOI:** 10.3390/ijerph18105349

**Published:** 2021-05-17

**Authors:** Sou Hyun Jang, JaHyun Kang

**Affiliations:** 1 Department of Sociology and Convergence Program for Social Innovation, Sungkyunkwan University, Seoul 03060, Korea; souhyunjang@skku.edu; 2 College of Nursing and Research Institute of Nursing Science, Seoul National University, Seoul 03080, Korea

**Keywords:** influenza vaccines, nativity, race, ethnicity

## Abstract

To support implementation strategies for upcoming influenza (flu) vaccinations for foreign-born and racial/ethnic minority groups, we analyzed the 2018 National Health Interview Survey (NHIS) database and performed logistic regression to examine the factors associated with, and the interaction between nativity and race/ethnicity in directing flu vaccination rates during the past 12 months (*n* = 25,045). As a result, we found nativity and race/ethnicity were associated with flu vaccination rates; foreign-born and non-Hispanic black respondents were less likely to take the vaccine than U.S.-born and non-Hispanic white respondents. The odds ratios were largest for the elderly, those working in the healthcare industry, those with health insurance, and those with a usual source of care (ORs = 3.058, 2.871, 2.317, and 2.342, respectively), suggesting that access to healthcare resources is critical for the uptake of the flu vaccine. There was a significant interaction effect between nativity and race/ethnicity. For improving flu vaccination rates, more support is necessary for foreign-born people and racial/ethnic minorities who have lower health insurance rates and usual sources of care than U.S.-born people and non-Hispanic whites, and thus are less able to adequately access healthcare resources in a timely manner.

## 1. Introduction

Seasonal influenza (flu) causes disease and socioeconomic burdens to individuals, as well as the healthcare system (e.g., symptomatic illnesses, medical visits, hospitalization, and death), and the burdens could be global, beyond usual society [1,2,3]. In the United States (U.S.), about 400,000 people were hospitalized, and about 22,000 died due to seasonal flu in the 2019–2020 season [4]. Globally, about 250,000 to 500,000 people died annually because of seasonal flu [5]. To prevent seasonal flu, the World Health Organization (WHO) [6] and the Centers for Disease and Control (CDC) [7] strongly recommend that people receive a flu vaccination, nationally targeting a vaccination rate of 70% among all age groups in the U.S. [8,9]. Nonetheless, on average, less than half of American adults have received a flu vaccine; for example, only 45.3% received one during the 2018–2019 flu season [10]. The benefits of flu vaccinations in the U.S. were estimated to be the prevention of 4.4 million flu illnesses, 58,000 flu hospitalizations, and 3500 flu deaths during the 2018–2019 flu season [7]. Racial/ethnic minorities, especially non-Hispanic Blacks, Hispanics, and non-Hispanic American Indians or Alaska Natives were found to have more burdens from the flu compared to non-Hispanic Whites [11]. For example, when 38 per 100,000 non-Hispanic Whites were hospitalized due to the flu, 68 non-Hispanic Blacks and 44 Hispanics were hospitalized. 

Previous studies have found that the rate of flu vaccination differs by nativity and race/ethnicity [12,13,14]. In general, more U.S.-born than foreign-born individuals [13] and more non-Hispanic Whites than non-Hispanic Blacks and Hispanics [14] took the flu vaccine. Considering nativity and Hispanic ethnicity, foreign-born Hispanics were less likely to take the flu vaccine than their U.S.-born and non-Hispanic White counterparts [14]. However, flu vaccination behaviors are less known and associated factors among non-Hispanic Asians and other racial/ethnic minority groups in comparison with non-Hispanic Whites. Barriers to healthcare services, such as health coverage, language barriers, concerns, and mistrust contribute to low flu vaccination rates among foreign-born and racial/ethnic minority groups [14,15]. Despite these findings, to the best of our knowledge, no study has examined the flu vaccination rate in light of nativity and race/ethnicity, encompassing all racial and ethnic groups, as well as reflecting CDC recommendations for flu vaccinations (in particular, among the elderly and healthcare personnel), the presence of health insurance, and the type of primary work industries. To support implementation strategies for upcoming flu vaccinations for foreign-born and racial/ethnical groups, this study aimed to compare the flu vaccination rates by nativity and race/ethnicity, and to examine associated factors and any interaction effect between nativity and race/ethnicity with regard to flu vaccinations.

## 2. Materials and Methods

### 2.1. Data 

In October 2020, we analyzed the most recent 2018 National Health Interview Survey (NHIS) data, which are nationally representative, cross-sectional household interview survey data including non-institutionalized U.S. residents [16]. There were 72,831 survey participants in the original data set. Since the factors associated with the uptake of the flu vaccine among adults and children/adolescents are known to be different, as the latter’s uptake is likely to be influenced by the perception of parents and healthcare providers [17], we excluded those who were under 18 years of age (*n* = 16,666). The 2018 NHIS data asked several questions about flu vaccinations in the past 12 months, including administration routes (i.e., injection vs. nasal spray). We excluded those who gave invalid answers, such as “refused”, “not ascertained”, and “did not know” (*n* = 31,120). Finally, we included a total of 25,045 respondents for this study. 

### 2.2. Measures 

The dependent variable of this study was the uptake of the flu vaccine in the past 12 months (no vs. yes). The independent variables were nativity (U.S.-born vs. foreign-born) and race/ethnicity (non-Hispanic Whites, non-Hispanic Blacks, Hispanics, non-Hispanic Asians, and non-Hispanic others). 

As covariates, we included the following variables: age (18–49, 50–64, and ≥65 years old), sex (male vs. female), education (below high school, high school graduate, some college, college graduate, and graduate education), marital status (unmarried vs. married), employment status (unemployed vs. employed), industry of primary work (healthcare, agriculture and mining, construction, utilities and manufacturing, wholesale and retail trade, transportation and warehousing, information, finance and insurance, real estate and rental leasing, services, public administration, and armed forces), health insurance (uninsured vs. insured), usual source of care (no vs. yes), self-rated health status (excellent/very good vs. good/fair/poor), and poverty level (at or above vs. below). We also included years of residence in the U.S. (<10 years vs. ≥10 years) as a proxy for assimilation for foreign-born participants. 

### 2.3. Statistical Analyses 

We used the chi-squared test to determine the differences in flu vaccination uptake by nativity and race/ethnicity. Logistic regression analysis was used to examine whether the relationship between flu vaccination uptake and nativity and race/ethnicity remained after controlling for covariates. We also tested interactions between nativity and race/ethnicity to examine whether there exists any moderation effect of nativity on the relationship between flu vaccination uptake and race/ethnicity. We used Stata/SE 16.1 (StataCorp LLC, College Station, TX, USA) for statistical analysis, and *p* < 0.05 was set for a significance level.

## 3. Results

The participants’ demographic characteristics are summarized in Table 1. Their mean age was 51.72 years (SD = 18.33), and the elderly (≥65 years) accounted for 28.8% of the study sample. More than half were female (54.5%) and unmarried (54.6%). Most (84.25%) were born in the U.S., and non-Hispanic White (68.39%) was the major racial/ethnic group, followed by Hispanics (12.52%), non-Hispanic Blacks (11.10%), non-Hispanic Asians (5.02%), and non-Hispanic others (2.97%). Slightly more than a third (35.46%) had a high school education or lower, while the rest had a college education or higher. More than half (55.44%) were employed, 20.37% worked in the service industry, and 7.10% primarily worked in the healthcare industry, including ambulatory healthcare services, hospitals, nursing, and residential care facilities. Most (88.22%) were at or above the poverty level. Most had health insurance (90.88%) and a usual source of health care (87.64%). Slightly more than half (58.1%) reported excellent or very good health. Among foreign-born participants, 82.18% had lived in the U.S. for 10 years or longer. 

The survey respondents who took the flu vaccine and those who did not differ in several demographic characteristics. The respondents who took the flu vaccine were more likely to be elderly, female, U.S.-born, non-Hispanic White, have some college education, be unemployed, be working in the healthcare industry (among employed), be at or above poverty level, insured, have a usual source of care, have good/fair/poor health status, and have lived in the U.S. for 10 years or longer (among those foreign-born).

**Figure 1:**
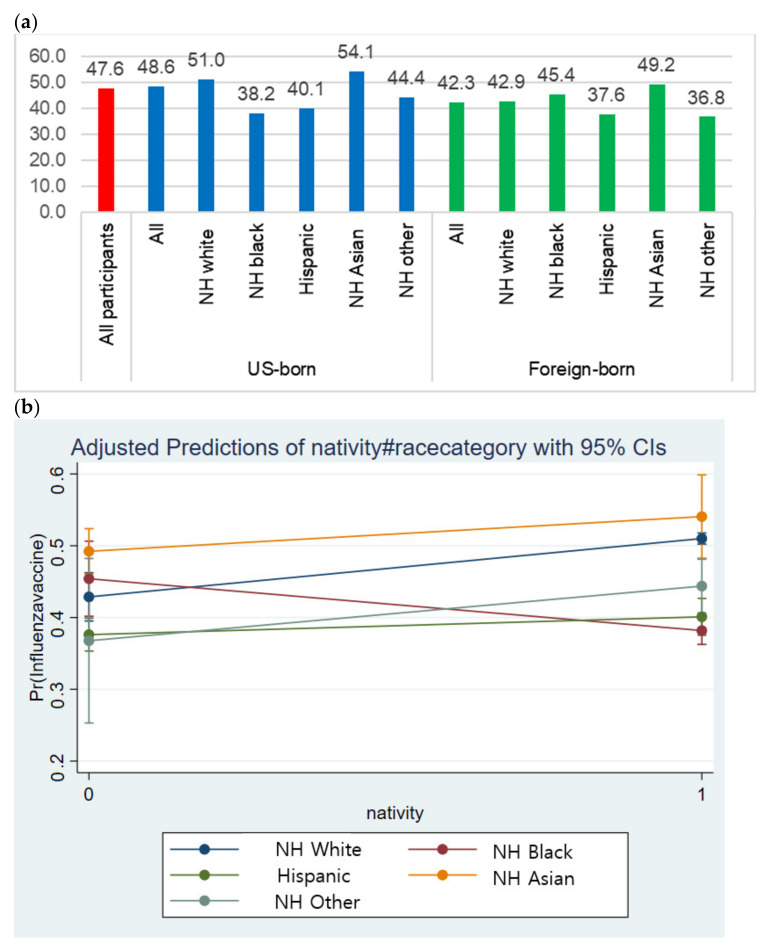
Influenza vaccination uptake and moderation effect of nativity. (**a**) Influenza vaccination uptake by nativity and race/ethnicity (%); The differences in Figure 1a were statistically significant based on chi-squared tests (*p* =0.000); (**b**) Moderation effect of nativity on the relationship between influenza vaccination uptake and race/ethnicity.

The flu vaccination rates were significantly different according to nativity and race/ethnicity (Figure 1a). While the average uptake rate was 47.6%, U.S.-born participants showed a higher vaccine uptake rate (48.6%) than the foreign-born participants (42.3%). Regardless of the nativity, non-Hispanic Asians showed higher vaccine uptake rates than any other racial/ethnic group. Among the U.S.-born respondents, non-Hispanic Blacks (38.2%), Hispanics (40.1%), and non-Hispanic others (44.4%) showed lower uptake rates than non-Hispanic Whites (51.0%). Foreign-born participants presented a different pattern; non-Hispanic Blacks reported a higher uptake rate (45.4%) than non-Hispanic Whites (42.9%), Hispanics (37.6%), and non-Hispanic others (36.8%). 

Nativity and race/ethnicity were significantly associated with flu vaccination uptake (Table 2). The participants who were born in the U.S. (odds ratio [OR] = 1.195; 95% confidence interval [CI] = 1.083–1.318) were more likely to receive the vaccine than those born in foreign countries. In addition, non-Hispanic Blacks (OR = 0.685; 95% CI = 0.623–0.753) were less likely to receive the vaccine than non-Hispanic whites. In addition to nativity and race/ethnicity, participants who were older (≥65 years and 50–64 years old [U.S.-born only]), female, married, with a college education or higher, working in the healthcare industry, insured, and with a usual source of care were more likely to receive the vaccine than their counterparts who were younger, male, unmarried, with lower education levels, working in other industries, uninsured, and without a usual source of care. The participants who were below the poverty level and reported excellent/very good health status were less likely to take flu vaccine than their counterparts who were at or above the poverty level and had good/fair/poor health status. The odds ratios were largest for the elderly, those working in the healthcare industry, those with health insurance, and those with usual sources of care (ORs = 3.058, 2.871, 2.317, and 2.342, respectively), suggesting that healthcare resources are critical for the uptake of the flu vaccine.

For both U.S.-born and foreign-born participants, the elderly, being non-Hispanic Asian, married, having a graduate education, working in the healthcare industry, having health insurance, and having a usual source of care were commonly and positively associated with flu vaccination uptake. Nevertheless, the associated factors also differed by participants’ nativity. Among U.S.-born participants, being 50–64 years old, being female, being a college graduate, and having a good/fair/poor self-reported health status were positively related to flu vaccination uptake, while a non-Hispanic Black ethnicity was negatively, and non-Hispanic Asian ethnicity was positively related to vaccination uptake. However, not all of these factors were significantly associated with foreign-born participants’ uptake. Instead, being Hispanic was positively related to the uptake among foreign-born participants. 

Being elderly, working in the healthcare industry, and having a usual source of care were commonly associated with flu vaccination across all racial and ethnic groups (Table 3). Yet, factors associated with flu vaccination uptake differed by respondents’ race and ethnicity. The factors which had the strongest impact on flu vaccination uptake also differed by different racial/ethnic groups, such as health insurance (non-Hispanic white [OR = 3.113, 95% CI = 2.583–3.750]), older age (non-Hispanic black [OR = 3.114, 95% CI = 2.439–3.976]), working in the healthcare industry (non-Hispanic Asian [OR = 4.200, 95% CI = 2.400–7.350], and non-Hispanic other ethnicity [OR = 3.445, 95% CI = 1.651–7.186]). 

Being female, having some college education, being below the poverty level, and excellent/very good self-reported health status were positively associated with flu vaccination uptake only among White respondents. Working in particular industries (i.e., agriculture, mining, utilities and manufacturing, and real estate and rental leasing) was negatively related to vaccine uptake among Whites. While being U.S.-born was positively related to flu vaccination uptake among non-Hispanic White (OR =1.619, 95% CI = 1.382–1.897) and non-Hispanic Asian respondents (OR = 1.587, 95% CI = 1.147–2.195), it was negatively related to vaccine uptake among non-Hispanic Black respondents (OR = 0.646, 95% CI = 0.493–0.847). Married status was positively related to vaccination uptake in all racial/ethnic groups, except the Hispanic group. Graduate education was positively related to flu vaccination uptake among other racial/ethnic groups, but not among non-Hispanic Asian and non-Hispanic other groups. Only among non-Hispanic other respondents, lower education levels were negatively related to flu vaccination uptake, such as high school graduates (OR = 0.485, CI = 0.265–0.888) and those with only some college education (OR = 0.503, CI = 0.277–0.913). Being 50–64 years old was positively related to vaccination uptake among all other racial/ethnic groups, but not significantly related to vaccination uptake among non-Hispanic Asian respondents.

While participants’ nativity and race/ethnicity were significantly associated with the vaccination uptake rate, the interaction between them was also statistically significant in predicting the uptake. Figure 1b presents the predictive margins of the interaction effects between nativity and race/ethnicity. Being born in the U.S. was positively related to increased flu vaccination uptake in general, but the effect pattern appeared to differ according to race/ethnicity. The line generated by immigrants’ race/ethnicity (non-Hispanic blacks) was not parallel to that generated by those born in the US, suggesting a moderation effect between the two factors—the positive impact of being born in the U.S. was found among all racial/ethnic groups, except for non-Hispanic Blacks.

## 4. Discussion

In line with the results of previous studies [14,18], this study found disparities in flu vaccination rates by nativity and race/ethnicity. In general, foreign-born and racial/ethnic minority groups showed lower rates of vaccination than their U.S.-born and non-Hispanic White counterparts, except for non-Hispanic Asians, regardless of nativity. Asian-American ethnicity, higher income [19], and educational attainment [20] were likely to contribute to a higher flu vaccination uptake rate. 

We also found that nativity and race/ethnicity interacted in regard to individuals’ flu vaccination uptake rates. While all U.S.-born participants reported higher uptake rates than foreign-born participants, there was no foreign-born disadvantage observed among non-Hispanic Blacks. This is similar to previous studies that commonly found a foreign-born advantage for Black adults in terms of the prevalence of diabetes [21] and disability [22]. Their higher level of English proficiency than other foreign-born racial/ethnic groups [23] and higher educational attainment levels and household income than U.S.-born Blacks [23] might have contributed to the foreign-born advantage in relation to flu vaccination among Blacks. In our dataset, we could confirm that foreign-born non-Hispanic Blacks had higher education levels and lower health insurance coverage than U.S.-born respondents (results were not presented). However, we could not verify their income level comparison due to the lack of income information in the dataset. The disparity between education levels and health insurance coverage among foreign-born non-Hispanic Blacks may be due to cultural norm differences between U.S.-born Hispanic Blacks and foreign-born Hispanic Blacks. We found that marital status was positively related to vaccination uptake in all racial/ethnic groups, except the Hispanic group. Although it was not feasible to examine why marital status was not associated with flu vaccination among Hispanics using data from a quantitative survey, the Hispanic culture, especially familism, might have influenced this finding. In other words, Hispanic respondents might be positively influenced to take the flu vaccine by their family members (including extended family members); thus, the role/impact of spouses might be weak. Compared to Hispanics, non-Hispanic respondents might be less influenced and supported by other family members than spouses, making one’s marital status a significant influence on flu vaccination behavior. Further studies are necessary to examine cultural norms and influences to understand these phenomena. Moreover, receiving or refusing vaccination is a social behavior influenced by social norms [24]. Engaging community partnerships (e.g., opinion leaders, faith-based organizations) with an effective outreach to specific race/ethnic minority groups would be an option to promote flu vaccination [25,26]. In addition, appropriately tailored social norm messages for these groups through online ethnic communities [27,28] or bilingual websites [29] could be another option for delivering health information to improve flu vaccination rates, as previous studies have found that they seek and share health information via these platforms.

On the other hand, one cross-sectional study on the minority community reported that one-third of U.S.-born Black participants did not intend to receive the vaccine despite the high risk of flu-related morbidity in the underserved minority populations [30]. U.S.-born Blacks may still be skeptical about receiving vaccines due to their history of medical research exploitation (e.g., the Tuskegee syphilis study) [31].

In addition to nativity and race/ethnicity, healthcare resources—including with regard to the elderly (in terms of having Medicare coverage), working in the healthcare industry, having health insurance, and having a usual place of care—had a strong and positive impact on flu vaccination uptake. In particular, the elderly, who were likely to be unemployed and who usually have Medicare coverage (i.e., if qualified, Medicare Part B covers annual flu vaccinations with no co-pay) [32], showed a higher flu vaccination uptake rate, as the CDC recommends vaccination for them as one of the high-risk groups [33]. In addition, as the CDC highly recommends the annual flu vaccination for healthcare personnel, they showed vaccination rates of 77.3–81.1% in the past 5 years [34]; our results confirmed that people working in the healthcare industry had higher flu uptake rates. Healthcare personnel might also have better knowledge/awareness of the importance of the flu vaccine and higher levels of self-motivation, as well as compliance with the mandatory flu vaccination policy [35] that may contribute to a higher vaccination rate than those who work in other industries. Interestingly, while we were analyzing the data, the significant differences in vaccination rates according to race/ethnicity (i.e., Hispanic and non-Hispanic Asians were more likely to receive a flu vaccination) disappeared, except for among non-Hispanic Blacks after adding the variable of primary work industry. Using the same data source (i.e., the NHIS data), Lu et al. reported a 64.8% flu vaccination rate among healthcare personnel and a significantly lower rate among non-Hispanic Blacks and Hispanics than among non-Hispanic Whites during the 2015–16 flu season [36]. If they added primary work industries in their model, the significantly lower rate of vaccination among the Hispanics might disappear, similar to our result. In any case, accessibility to healthcare services seems to be a critical factor for flu vaccination uptake for most of the races/ethnicities.

While a 70% flu vaccination rate was one of the goals of the U.S. Department of Health and Human Services’ Healthy People 2020 and 2030 campaign [9], persistent gaps have remained between the goals and actual vaccination rates of racial and ethnic minority populations [37]. Multiple factors are attributed to this suboptimal coverage phenomenon, including vaccine misconceptions, skepticism about annual flu vaccinations, efficacy/safety concerns, lack of knowledge, and perceived risks [37]. Thus, to improve flu vaccination uptake rates in racial/ethnic minority groups and reduce the persistent gaps, further studies are necessary to examine any reasons/biases behind low vaccination uptake minority groups and suggest a customized strategy for each group.

This study has several limitations. First, although we used the most recently available NHIS data, it was collected in the pre-COVID-19 period. Therefore, the associated factors found in this study might differ from the associated factors with flu vaccinations during the COVID-19 pandemic. As the flu epidemic was found to be lower than usual during the COVID-19 pandemic [38], future studies could examine the factors associated with flu vaccinations during COVID-19, focusing on foreign-borns and racial and ethnic minorities who are more vulnerable during the pandemic [31]. In addition to flu vaccinations, future studies could also examine the factors associated with COVID-19 vaccinations, especially focusing on minority groups who might also be less likely to get vaccinated due to their vulnerability. Second, we did not include household income in our statistical analysis due to its many missing values. However, we believe that other sociodemographic characteristics that are related to income, such as educational attainment or poverty level, could remedy this limitation. Third, we were not able to specify the ethnicity, except for Hispanic ethnicity, because the NHIS data did not provide ethnicity data. In fact, there exists a diversity in health status by the country of origin within the Hispanic [39] and Black communities [40]. A previous study [14] found disparities in flu vaccination uptake rates among Asian-American subgroups (e.g., Filipino and Japanese). Considering that racial subgroups are not homogeneous, future studies need to examine the disparities in, and factors associated with flu vaccination uptake among different subgroups by country of origin. Fourth, we were not able to examine individuals’ perception of flu vaccination, which is known to be one of the crucial factors to the uptake, especially during the pandemic [41]. In addition, we could not examine any reasons/biases or socio-cultural norms behind vaccine hesitancy among the minority groups due to the limited information in the NHIS. Lastly, because NHIS flu vaccination uptake responses were not verified with vaccination records, our results may have presented a higher vaccination uptake rate similar to a prior study that reported a potential response bias where more respondents had health coverage than people who did not participate in the survey [18].

## 5. Conclusions

To improve flu vaccination rates, there needs to be more support for people, especially foreign-born people and racial/ethnic minorities who have a lower health insurance rate and less sources of care than U.S.-born people and non-Hispanic Whites [42,43], so that they can access healthcare resources in a timely and adequate manner. To meet the “Healthy People 2030” campaign’s goal of a 70% flu vaccination rate among Americans and to reduce individual and societal burdens, encouraging flu vaccination among racial/ethnic minority groups through tailored social messages would be more important.

## Figures and Tables

**Table 1:** Demographic characteristics of survey participants.

	All (*n* = 25,045)	Did Not Receive Influenza Vaccine (*n* = 13,121)	Received Influenza Vaccine (*n* = 11,924)	*p*-Value
Age				0.000
Mean (SD)	51.72 (18.33)	46.87 (17.06)	57.04 (18.20)	
18–49 years	11,355 (45.34)	7348 (56.00)	4007 (33.60)	
50–64 years	6477 (25.86)	3468 (26.43)	3009 (25.23)	
65 years or older	7213 (28.80)	2305 (17.57)	4908 (41.16)	
Sex				0.000
Male	11,396 (45.50)	6461 (49.24)	4935 (41.39)	
Female	13,649 (54.50)	6660 (50.76)	6989 (58.61)	
Marital status				0.000
Unmarried	13,645 (54.60)	7642 (58.40)	6003 (50.42)	
Married	11,346 (45.40)	5444 (41.60)	5902 (49.58)	
Nativity				0.000
US-born	21,061 (84.25)	10,819 (82.64)	10,242 (86.03)	
Foreign-born	3936 (15.75)	2273 (16.36)	1663 (13.97)	
Race/ethnicity				0.000
Non-Hispanic white	17,129 (68.39)	8460 (64.48)	8669 (72.70)	
Non-Hispanic black	2779 (11.10)	1692 (12.90)	1087 (9.12)	
Hispanic	3136 (12.52)	1922 (14.65)	1214 (10.18)	
Non-Hispanic Asian	1257 (5.02)	628 (4.79)	629 (5.28)	
Non-Hispanic others	744 (2.97)	419 (3.19)	325 (2.73)	
Education				0.000
Below high school	2268 (9.09)	1271 (9.73)	997 (8.39)	
High school graduate	6579 (26.37)	3730 (28.54)	2849 (23.98)	
Some college	7552 (30.27)	4169 (31.90)	3383 (28.47)	
College graduate	5340 (21.40)	2645 (20.24)	2695 (22.68)	
Graduate education	3212 (12.87)	1254 (9.60)	1958 (16.48)	
Employment status and work industry				0.000
Unemployed	11,156 (44.56)	4863 (37.40)	6293 (53.04)	
Employed work industry				
Healthcare	1766 (7.10)	540 (4.15)	1226 (10.33)	
Agriculture, mining	248 (1.00)	170 (1.31)	78 (0.66)	
Construction	897 (3.61)	682 (5.25)	215 (1.81)	
Utilities and manufacturing	1419 (5.71)	887 (6.82)	532 (4.48)	
Wholesale and retail trade	1638 (6.59)	1117 (8.59)	521 (4.39)	
Transportation and warehousing	617 (2.48)	440 (3.38)	177 (1.49)	
Information	272 (1.09)	153 (1.18)	119 (1.00)	
Finance and insurance	705 (2.83)	384 (2.95)	320 (2.70)	
Real estate and rental leasing	312 (1.25)	214 (1.65)	98 (0.83)	
Services	5066 (20.37)	3160 (24.31)	1906 (16.07)	
Public administration and armed forces	770 (3.10)	391 (3.03)	379 (3.19)	
Poverty level				0.000
At or above	21,099 (88.22)	10,867 (86.43)	10,232 (90.21)	
Below	2817 (11.78)	1706 (13.57)	1111 (9.79)	
Health insurance				0.000
Uninsured	2278 (9.12)	1893 (14.48)	385 (3.23)	
Insured	22,693 (90.88)	11,176 (85.52)	11,517 (96.77)	
Usual source of care				0.000
No	3096 (12.36)	2470 (18.83)	626 (5.25)	
Yes	21,945 (87.64)	10,648 (81.17)	11,297 (94.75)	
Self-reported health				0.000
Good/fair/poor	10,491 (41.90)	5148 (39.25)	5343 (44.82)	
Excellent/very good	14,546 (58.10)	7967 (60.75)	6579 (55.18)	
Years in the U.S. (among foreign-born)				0.000
10 years or longer	3218 (82.18)	1795 (79.50)	1423 (85.83)	
<10 years	698 (17.82)	463 (20.50)	235 (14.17)	

The figures are numbers (percentages) unless otherwise noted. The following variables have missing values: nativity (*n* = 48), marital status (*n* = 54), education (*n* = 94), employment status (*n* = 11), poverty level (*n* = 1129), health insurance status (*n* = 74), usual place of care (*n* = 4), and self-rated health status (*n* = 8). The chi-squared test was used to determine the statistical difference between those who received the influenza vaccine and those who did not.

**Table 2:** Factors associated with influenza vaccine uptake by nativity.

	All	US-Born	Foreign-Born
Age			
18–49 years (ref)	1.0	1.0	1.0
50–64 years	1.436 (1.339–1.539 ***	1.490 (1.381–1.608) ***	1.173 (0.977–1.408)
65 years or older	3.058 (2.823–3.312) ***	3.135 (2.873–3.419) ***	2.539 (2.039–3.161) ***
Sex			
Male (ref)	1.0	1.0	1.0
Female	1.125 (1.061–1.192) ***	1.150 (1.079–1.225) ***	1.016 (0.872–1.183)
Nativity			
Foreign-born (ref)	1.0	-	-
U.S.-born	1.195 (1.083–1.318) ***	-	-
Race/ethnicity			
Non-Hispanic white (ref)	1.0	1.0	1.0
Non-Hispanic black	0.685 (0.623–0.753) ***	0.634 (0.573–0.701) ***	1.316 (0.985–1.758)
Hispanic	1.063 (0.959–1.178)	0.978 (0.861–1.110)	1.298 (1.057–1.594) *
Non-Hispanic Asian	1.148 (0.991–1.331)	1.346 (1.029–1.762) *	1.427 (1.157–1.760) ***
Non-Hispanic others	1.036 (0.876–1.224)	1.044 (0.875–1.245)	1.237 (0.703–2.176)
Marital status			
Unmarried (ref)	1.0	1.0	1.0
Married	1.216 (1.147–1.289) ***	1.211 (1.136–1.291) ***	1.168 (1.006–1.355) *
Education			
Below high school (ref)	1.0	1.0	1.0
High school graduate	0.961 (0.858–1.077)	0.999 (0.874–1.141)	0.945 (0.752–1.187)
Some college	1.025 (0.914–1.148)	1.093 (0.958–1.248)	0.851 (0.666–1.088)
College graduate	1.394 (1.234–1.575) ***	1.525 (1.323–1.757) ***	1.032 (0.800–1.332)
Graduate education	1.954 (1.709–2.234) ***	2.139 (1.831–2.498) ***	1.501 (1.131–1.991) **
Employment status			
Unemployed (ref)	1.0	1.0	1.0
Employed work industry			
Healthcare	2.871 (2.537–3.250) ***	2.818 (2.459–3.229) ***	3.039 (2.235–4.132) ***
Agriculture, mining	0.649 (0.484–0.869) **	0.589 (0.426–0.816) ***	1.000 (0.508–1.966)
Construction	0.498 (0.417–0.594) ***	0.470 (0.387–0.570) ***	0.611 (0.397–0.939) *
Utilities and manufacturing	0.775 (0.682–0.881) ***	0.787 (0.684–0.906) ***	0.723 (0.529–0.988) *
Wholesale and retail trade	0.626 (0.553–0.708) ***	0.627 (0.549–0.717) ***	0.600 (0.423–0.851) **
Transportation and warehousing	0.585 (0.481–0.710) ***	0.543 (0.437–0.674) ***	0.763 (0.487–1.196)
Information	0.871 (0.672–1.128)	0.927 (0.799–1.229)	0.623 (0.315–1.232)
Finance and insurance	0.885 (0.749–1.045)	0.873 (0.729–1.047)	0.930 (0.601–1.439)
Real estate and rental leasing	0.474 (0.364–0.616) ***	0.458 (0.343–0.612) ***	0.600 (0.314–1.144)
Services	0.705 (0.648–0.766) ***	0.684 (0.624–0.750) ***	0.769 (0.628–0.942) *
Public administration and armed forces	1.002 (0.853–1.175)	0.984 (0.830–1.166)	1.115 (0.675–1.843)
Poverty level			
At or above (ref)	1.0	1.0	1.0
Below	0.983 (0.894–1.082)	0.960 (0.861–1.069)	1.102 (0.895–1.356)
Health insurance			
Uninsured (ref)	1.0	1.0	1.0
Insured	2.317 (2.041–2.629) ***	2.682 (2.303–3.124) ***	1.802 (1.416–2.293) ***
Usual source of care			
No (ref)	1.0	1.0	1.0
Yes	2.342 (2.112–2.599) ***	2.373 (2.112–2.666) ***	2.367 (1.872–2.993) ***
Self-reported health			
Good/fair/poor (ref)	1.0	1.0	1.0
Excellent/very good	0.865 (0.814–0.919) ***	0.838 (0.785–0.895) ***	0.988 (0.844–1.157)
Years in the U.S. (foreign-born only)			
10 years or longer	-	-	1.0
<10 years	-	-	0.969 (0.789–1.190)
Cons	0.100 (0.081–0.122) ***	0.098 (0.079–0.121) ***	0.124 (0.082–0.187) ***
Pseudo R^2^	0.1216	0.1263	0.1049
*N*	23,616	19.951	3,643

The figures are odds ratios (confidence intervals). The following variables have missing values: nativity (*n* = 48), marital status (*n* = 54), education (*n* = 94), employment status (*n* = 11), poverty level (1,129), health insurance status (*n* = 74), usual place of care (*n* = 4), and self-rated health status (*n* = 8). *** *p* < 0.001; ** *p* < 0.01; * *p* < 0.05.

**Table 3:** Factors associated with influenza vaccine uptake by race and ethnicity.

	NH White	NH Black	Hispanic	NH Asian	NH Others
Age					
18–49 years (ref)	1.0	1.0	1.0	1.0	1.0
50–64 years	1.437 (1.321–1.562) ***	1.612 (1.301–1.998) ***	1.306 (1.063–1.605) *	1.352 (0.962–1.900)	1.632 (1.082–2.460) *
65 years or older	3.076 (2.796–3.384) ***	3.114 (2.439–3.976) ***	2.728 (2.115–3.519) ***	3.333 (2.267–4.900) ***	2.505 (1.548–4.053) ***
Sex					
Male (ref)	1.0	1.0	1.0	1.0	1.0
Female	1.150 (1.072–1.233) ***	0.928 (0.772–1.114)	1.183 (0.994–1.409)	1.130 (0.871–1.465)	1.232 (0.872–1.741)
Nativity					
Foreign-born (ref)	1.0	1.0	1.0	1.0	1.0
U.S.-born	1.619 (1.382–1.897) ***	0.646 (0.493–0.847) **	1.063 (0.891–1.267)	1.587 (1.147–2.195) **	1.307 (0.711–2.402)
Marital status					
Unmarried (ref)	1.0	1.0	1.0	1.0	1.0
Married	1.169 (1.091–1.253) ***	1.246 (1.018–1.526) *	1.150 (0.973–1.358)	1.461 (1.104–1.933) **	1.639 (1.144–2.348) **
Education					
Below high school (ref)	1.0	1.0	1.0	1.0	1.0
High school graduate	1.079 (0.920–1.266)	1.171 (0.875–1.566)	0.871 (0.691–1.098)	0.777 (0.421–1.433)	0.485 (0.265–0.888) *
Some college	1.215 (1.037–1.424) *	1.224 (0.906–1.653)	0.817 (0.638–1.045)	0.623 (0.340–1.142)	0.503 (0.277–0.913) *
College graduate	1.668 (1.414–1.969) ***	1.484 (1.041–2.116) *	1.056 (0.787–1.419)	0.880 (0.492–1.574)	0.759 (0.378–1.525)
Graduate education	2.367 (1.981–2.829) ***	1.778 (1.191–2.653) **	1.699 (1.163–2.481) **	1.160 (0.632–2.129)	1.262 (0.561–2.837)
Employment status					
Unemployed (ref)	1.0	1.0	1.0	1.0	1.0
Employed work industry					
Healthcare	2.926 (2.500–3.424) ***	2.452 (1.761–3.414) ***	2.522 (1.807–3.520) ***	4.200 (2.400–7.350) ***	3.445 (1.651–7.186) ***
Agriculture, mining	0.615 (0.437–0.865) **	0.425 (0.085–2.103)	0.997 (0.499–1.991)	0.754 (0.124–4.562)	0.543 (0.052–5.645)
Construction	0.484 (0.394–0.594) ***	0.169 (0.050–0.574) **	0.576 (0.366–0.904) *	0.526 (0.183–1.151)	0.994 (0.387–2.549)
Utilities and manufacturing	0.748 (0.642–0.871) ***	0.749 (0.475–1.179)	0.856 (0.594–1.235)	0.962 (0.656–1.636)	1.036 (0.430–2.494)
Wholesale and retail trade	0.604 (0.522–0.599) ***	0.819 (0.532–1.257)	0.802 (0.565–1.140)	0.448 (0.251–0.799) **	0.439 (0.191–1.005)
Transportation and warehousing	0.505 (0.394–0.646) ***	0.480 (0.288–0.800) **	1.103 (0.634–1.918)	1.047 (0.489–2.242)	0.573 (0.194–1.692)
Information	0.882 (0.655–1.189)	0.929 (0.337–2.255)	0.812 (0.291–2.267)	0.756 (0.288–1.982)	1.271 (0.284–5.673)
Finance and insurance	0.894 (0.733–1.092)	0.620 (0.348–1.103)	1.019 (0.616–1.686)	1.010 (0.539–1.921)	1.484 (0.432–5.097)
Real estate and rental leasing	0.484 (0.358–0.655) ***	0.491 (0.189–1.277)	0.526 (0.238–1.162)	0.242 (0.049–1.182)	0.340 (0.027–4.169)
Services	0.693 (0.626–0.768) ***	0.644 (0.495–0.836) ***	0.814 (0.648–1.023)	0.740 (0.516–1.062)	0.608 (0.377–0.980)
Public administration and armed forces	1.003 (0.827–1.218)	0.850 (0.548–1.317)	1.153 (0.684–1.946)	1.251 (0.560–2.798)	1.122 (0.474–2.653)
Poverty level					
At or above (ref)	1.0	1.0	1.0	1.0	1.0
below	0.856 (0.750–0.977) *	1.110 (0.886–1.389)	1.136 (0.911–1.417)	1.165 (0.749–1.813)	1.059 (0.684–1.638)
Health insurance					
Uninsured (ref)	1.0	1.0	1.0	1.0	1.0
Insured	3.113 (2.583–3.750) ***	1.976 (1.369–2.852) ***	2.058 (1.601–2.645) ***	1.328 (0.727–2.423)	1.508 (0.922–2.466)
Usual source of care					
No (ref)	1.0	1.0	1.0	1.0	1.0
Yes	2.415 (2.119–2.752) ***	2.648 (1.863–3.765) ***	1.993 (1.553–2.557) ***	2.608 (1.713–3.970) ***	2.359 (1.328–4.190) **
Self-reported health					
Good/fair/poor (ref)	1.0	1.0	1.0	1.0	1.0
Excellent/very good	0.838 (0.779–0.902) ***	0.870 (0.722–1.049)	0.887 (0.746–1.054)	0.978 (0.733–1.305)	0.890 (0.626–1.265)
Cons	0.049 (0.036–0.066) ***	0.119 (0.067–0.210) ***	0.151 (0.104–0.219) ***	0.193 (0.080–0.464) ***	0.206 (0.071–0.591) **
Pseudo R^2^	0.1242	0.1097	0.1049	0.1286	0.1238
*N*	16,257	2579	2909	1169	702

The figures are odds ratios (confidence intervals). The following variables have missing values: nativity (*n* = 48), marital status (*n* = 54), education (*n* = 94), employment status (*n* = 11), poverty level (1129), health insurance status (*n* = 74), usual place of care (*n* = 4), and self-rated health status (*n* = 8). *** *p*<0.001; ** *p*<0.01; * *p*<0.05.
